# 2-[3-(1,3-Benzothia­zol-2-yl)-2,2-di­methyl­prop­yl]-2-methyl-2,3-di­hydro-1,3-benzothia­zole

**DOI:** 10.1107/S1600536812029297

**Published:** 2012-07-07

**Authors:** Sammer Yousuf, Hina Siddiqui, Rabia Farooq, M. Iqbal Choudhary

**Affiliations:** a H.E.J. Research Institute of Chemistry, International Center for Chemical and Biological Sciences, University of Karachi, Karachi 75270, Pakistan; b Department of Biochemistry, Faculty of Sciences, King Abdulaziz University, Jeddah 21589, Saudi Arabia

## Abstract

In the title compound, C_20_H_22_N_2_S_2_, the five-membered thia­zole ring of the 2-methyl-2,3-dihydro-1,3-benzothia­zole unit has an envelope conformation. The dihedral angle between the planar [maximum deviation of 0.014 (1) Å for the S atom] benzothia­zole ring system and the benzene ring is 78.37 (12)°. Two intra­molecular C—H⋯S hydrogen bonds are observed, forming rings of graph-set motif *S*(6). In the crystal, the molecules are consolidated in pairs through N—H⋯N hydrogen bonds and are arranged parallel to the *b* axis.

## Related literature

For the biological activity of benzothia­zoles, see: Prabhu *et al.* (2011 ▶); Chaudhary *et al.* (2010 ▶): Kaur *et al.* (2010 ▶). For the crystal structures of closely related compounds see: Ghalib *et al.* (2011 ▶); Chen *et al.* (2009 ▶); Brandenburg *et al.* (1987 ▶).
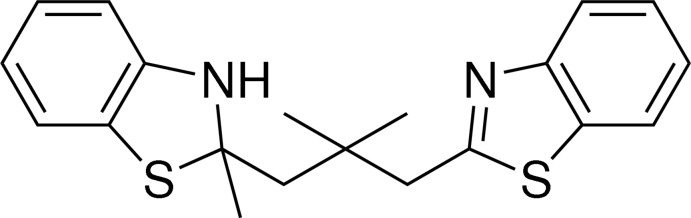


## Experimental

### 

#### Crystal data

C_20_H_22_N_2_S_2_
*M* *_r_* = 354.52
Triclinic, 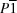

*a* = 9.8472 (8) Å
*b* = 9.9039 (8) Å
*c* = 11.7974 (9) Å
α = 88.490 (2)°
β = 67.006 (2)°
γ = 60.764 (2)°
*V* = 904.64 (12) Å^3^
*Z* = 2
Mo *K*α radiation
μ = 0.30 mm^−1^
*T* = 273 K
0.49 × 0.13 × 0.05 mm

#### Data collection

Bruker SMART APEX CCD area-detector diffractometer
Absorption correction: multi-scan (*SADABS*; Bruker, 2000 ▶) *T*_min_ = 0.868, *T*_max_ = 0.985
10184 measured reflections
3354 independent reflections
2667 reflections with *I* > 2σ(*I*)
*R*_int_ = 0.030

#### Refinement

*R*[*F* ^2^ > 2σ(*F* ^2^)] = 0.042
*wR*(*F* ^2^) = 0.113
*S* = 0.91
3354 reflections
224 parameters
2 restraints
H atoms treated by a mixture of independent and constrained refinement
Δρ_max_ = 0.39 e Å^−3^
Δρ_min_ = −0.16 e Å^−3^

### 

Data collection: *SMART* (Bruker, 2000 ▶); cell refinement: *SAINT* (Bruker, 2000 ▶); data reduction: *SAINT*; program(s) used to solve structure: *SHELXS97* (Sheldrick, 2008 ▶); program(s) used to refine structure: *SHELXL97* (Sheldrick, 2008 ▶); molecular graphics: *SHELXTL* (Sheldrick, 2008 ▶); software used to prepare material for publication: *SHELXTL*, *PARST* (Nardelli, 1995 ▶) and *PLATON* (Spek, 2009 ▶).

## Supplementary Material

Crystal structure: contains datablock(s) global, I. DOI: 10.1107/S1600536812029297/rz2780sup1.cif

Structure factors: contains datablock(s) I. DOI: 10.1107/S1600536812029297/rz2780Isup2.hkl

Supplementary material file. DOI: 10.1107/S1600536812029297/rz2780Isup3.cml

Additional supplementary materials:  crystallographic information; 3D view; checkCIF report

## Figures and Tables

**Table 1:** Hydrogen-bond geometry (Å, °)

*D*—H⋯*A*	*D*—H	H⋯*A*	*D*⋯*A*	*D*—H⋯*A*
N1—H1*N*2⋯N2^i^	0.79 (3)	2.35 (3)	3.130 (3)	170 (4)
C10—H10*B*⋯S1	0.97	2.87	3.543 (2)	128
C20—H20*B*⋯S1	0.96	2.67	3.331 (4)	126

Symmetry code: (i) 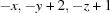.

**Fig. 1.**
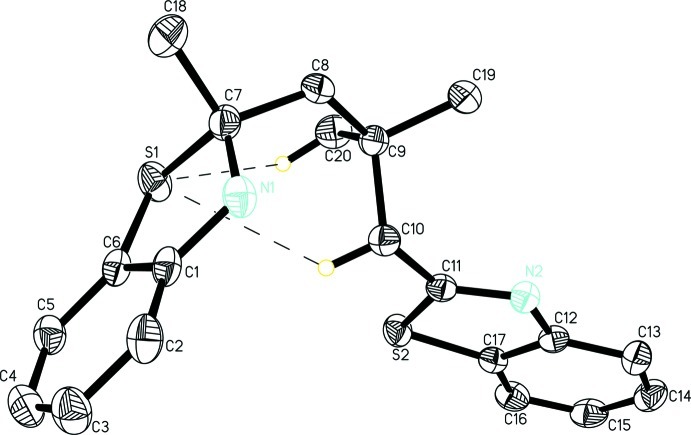
The molecular structure of the titke compound with displacement ellipsoids drawn at the 30% probability level. Hydrogen atoms not involved in intramolecular hydrogen bonds (dashed lines) are omitted.

**Fig. 2.**
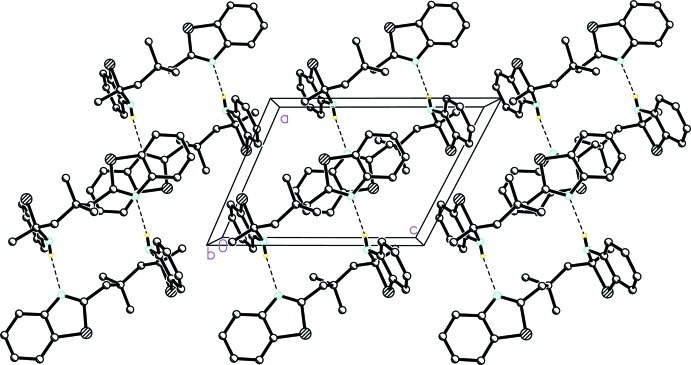
The crystal packing of the title compound viewed down the b axis. Only hydrogen atoms involved in hydrogen bonding (dashed lines) are shown.

## supplementary crystallographic information

### Comment

Benzothiazole derivatives have attracted remarkable interest in medicinal chemistry due to their diverse biological properties, such as analgesic, anti-viral, anti-bacterial, anti-inflammatory, anti-cancer, anti-diabetic and anti-HIV activities (Prabhu *et al.*, 2011; Chaudhary *et al.*, 2010; Kaur *et al.*, 2010). The title compound was an unexpected product obtained during the synthesis of different benzothiazoles derivatives to study their bioactive potential and to establish the structure-activity relationship (SAR).

The title compound (Fig. 1) is composed of a benzothiazole (S2/N2/C11–C17) and a 2,3-dihydrobenzothiazole (S1/N1/C1–C7) ring connected through a dimethyl propyl chain (C8–C10/C19/C20). The five membered dihydrobenzothiazole ring (S1/N1/C1/C6–C7) assumes an envelope conformation (*Q* = 0.252 (3) Å and φ = 321.5 (6)°) with the *pseudo* axially oriented methyl group attached at C7. The benzothiazole ring (S2/N2/C11–C17) is planar (maximum deviation of 0.014 (1) Å for atom S2) and forms a dihedral angle of 78.37 (12)° with the C1–C6 phenyl ring. The envelope conformation of the dihydrobenzothiazole ring is stabilized by two intramolecular C10–H10B···S1 and C20–H20B···S1 hydrogen interactions (Table 1) forming two *S*(6) ring motifs. In the crystal, the molecules are consolidated in pairs through N—H···N intermolecular hydrogen bonds and arranged parallel to the *b* axis (Fig. 2 and Table 1). Bond lengths and angles are within the normal range and similar to those reported for closely related structures (Ghalib *et al.*, 2011; Chen *et al.*, 2009; Brandenburg *et al.*, 1987).

### Experimental

A solution of dimedone (5,5-dimethylcyclohexane-1,3-dione; 500 mg, 3.5 mmol) and 2-aminothiophenol (0.384 ml, 3.5 mmol) in a mixture of acetic acid-ethanol (1:1 *v*/*v*, 15 ml) was stirred for 4 h. Progress of the reaction was monitored by thin layer chromatography in hexane:ethyl acetate (7:3 *v*/*v*) solvent system. After completion, the reaction mixture was dried under vacuum. Flash chromatography yielded the title compound in 55% yield (690 mg). Slow evaporation of an acetone/hexanes solution (1:1 *v*/*v*) yielded suitable crystals for single-crystal X-ray diffraction studies. All the starting material and solvents were purchased from commercial suppliers and used without purification.

### Refinement

C-bound H atoms were positioned geometrically with C—H = 0.93–0.96 Å and constrained to ride on their parent atoms with *U*_iso_(H) = 1.2*U*_eq_(C) or 1.5*U*_eq_(C) for methyl H atoms. The H atom on the nitrogen atom was located in a difference Fourier map and refined isotropically (N—H = 0.79 (3) Å). A rotating group model was applied to the methyl groups.

### Crystal data

**Table:**

C_20_H_22_N_2_S_2_	*Z* = 2
*M**_r_* = 354.52	*F*(000) = 376
Triclinic, *P*1	*D*_x_ = 1.301 Mg m^−^^3^
Hall symbol: -P 1	Mo *K*α radiation, λ = 0.71073 Å
*a* = 9.8472 (8) Å	Cell parameters from 2480 reflections
*b* = 9.9039 (8) Å	θ = 1.9–25.5°
*c* = 11.7974 (9) Å	µ = 0.30 mm^−^^1^
α = 88.490 (2)°	*T* = 273 K
β = 67.006 (2)°	Plate, yellow
γ = 60.764 (2)°	0.49 × 0.13 × 0.05 mm
*V* = 904.64 (12) Å^3^	

### Data collection

**Table:**

Bruker SMART APEX CCD area-detector diffractometer	3354 independent reflections
Radiation source: fine-focus sealed tube	2667 reflections with *I* > 2σ(*I*)
Graphite monochromator	*R*_int_ = 0.030
ω scan	θ_max_ = 25.5°, θ_min_ = 1.9°
Absorption correction: multi-scan (*SADABS*; Bruker, 2000)	*h* = −11→11
*T*_min_ = 0.868, *T*_max_ = 0.985	*k* = −11→11
10184 measured reflections	*l* = −14→14

### Refinement

**Table:**

Refinement on *F*^2^	Primary atom site location: structure-invariant direct methods
Least-squares matrix: full	Secondary atom site location: difference Fourier map
*R*[*F*^2^ > 2σ(*F*^2^)] = 0.042	Hydrogen site location: inferred from neighbouring sites
*wR*(*F*^2^) = 0.113	H atoms treated by a mixture of independent and constrained refinement
*S* = 0.91	*w* = 1/[σ^2^(*F*_o_^2^) + (0.0642*P*)^2^ + 0.3605*P*] where *P* = (*F*_o_^2^ + 2*F*_c_^2^)/3
3354 reflections	(Δ/σ)_max_ < 0.001
224 parameters	Δρ_max_ = 0.39 e Å^−^^3^
2 restraints	Δρ_min_ = −0.16 e Å^−^^3^

### Special details

**Table:**

Geometry. All e.s.d.'s (except the e.s.d. in the dihedral angle between two l.s. planes) are estimated using the full covariance matrix. The cell e.s.d.'s are taken into account individually in the estimation of e.s.d.'s in distances, angles and torsion angles; correlations between e.s.d.'s in cell parameters are only used when they are defined by crystal symmetry. An approximate (isotropic) treatment of cell e.s.d.'s is used for estimating e.s.d.'s involving l.s. planes.
Refinement. Refinement of *F*^2^ against ALL reflections. The weighted *R*-factor *wR* and goodness of fit *S* are based on *F*^2^, conventional *R*-factors *R* are based on *F*, with *F* set to zero for negative *F*^2^. The threshold expression of *F*^2^ > σ(*F*^2^) is used only for calculating *R*-factors(gt) *etc*. and is not relevant to the choice of reflections for refinement. *R*-factors based on *F*^2^ are statistically about twice as large as those based on *F*, and *R*- factors based on ALL data will be even larger.

### Fractional atomic coordinates and isotropic or equivalent isotropic displacement parameters (Å^2^)

**Table:**

	*x*	*y*	*z*	*U*_iso_*/*U*_eq_	
S1	0.32354 (9)	0.83415 (8)	0.06044 (6)	0.0508 (2)	
S2	0.60463 (8)	0.61184 (7)	0.35113 (6)	0.0445 (2)	
N1	0.0246 (3)	0.9353 (3)	0.2519 (2)	0.0436 (5)	
N2	0.3465 (2)	0.8013 (2)	0.55742 (18)	0.0379 (5)	
C1	0.0589 (3)	0.8023 (3)	0.1830 (2)	0.0385 (6)	
C2	−0.0418 (3)	0.7350 (3)	0.2115 (2)	0.0501 (7)	
H2B	−0.1497	0.7839	0.2801	0.060*	
C3	0.0203 (4)	0.5937 (4)	0.1367 (3)	0.0580 (8)	
H3A	−0.0469	0.5479	0.1558	0.070*	
C4	0.1790 (4)	0.5200 (4)	0.0346 (3)	0.0558 (7)	
H4A	0.2189	0.4245	−0.0138	0.067*	
C5	0.2793 (3)	0.5872 (3)	0.0039 (2)	0.0460 (6)	
H5A	0.3863	0.5382	−0.0657	0.055*	
C6	0.2192 (3)	0.7280 (3)	0.0774 (2)	0.0386 (6)	
C7	0.1258 (3)	1.0049 (3)	0.1824 (2)	0.0428 (6)	
C8	0.1569 (3)	1.0844 (3)	0.2717 (2)	0.0421 (6)	
H8A	0.0463	1.1468	0.3440	0.051*	
H8C	0.1845	1.1590	0.2293	0.051*	
C9	0.2906 (3)	0.9950 (3)	0.3245 (2)	0.0375 (6)	
C10	0.2804 (3)	0.8512 (3)	0.3731 (2)	0.0383 (6)	
H10A	0.1617	0.8876	0.4310	0.046*	
H10B	0.3125	0.7766	0.3024	0.046*	
C11	0.3927 (3)	0.7671 (3)	0.4378 (2)	0.0348 (5)	
C12	0.4823 (3)	0.7044 (3)	0.5878 (2)	0.0363 (5)	
C13	0.4762 (3)	0.7113 (3)	0.7076 (2)	0.0469 (6)	
H13A	0.3756	0.7846	0.7761	0.056*	
C14	0.6216 (4)	0.6076 (3)	0.7226 (3)	0.0532 (7)	
H14A	0.6184	0.6107	0.8024	0.064*	
C15	0.7724 (4)	0.4990 (3)	0.6216 (3)	0.0554 (7)	
H15A	0.8689	0.4303	0.6345	0.067*	
C16	0.7823 (3)	0.4906 (3)	0.5024 (3)	0.0502 (7)	
H16A	0.8843	0.4181	0.4344	0.060*	
C17	0.6351 (3)	0.5939 (3)	0.4866 (2)	0.0381 (6)	
C18	0.0321 (4)	1.1296 (4)	0.1175 (3)	0.0687 (9)	
H18A	−0.0691	1.2194	0.1799	0.103*	
H18B	−0.0010	1.0853	0.0691	0.103*	
H18C	0.1077	1.1621	0.0628	0.103*	
C19	0.2421 (4)	1.1127 (3)	0.4357 (3)	0.0502 (7)	
H19A	0.2371	1.2064	0.4081	0.075*	
H19B	0.3275	1.0654	0.4676	0.075*	
H19C	0.1315	1.1399	0.5009	0.075*	
C20	0.4729 (3)	0.9444 (3)	0.2260 (3)	0.0509 (7)	
H20A	0.4713	1.0324	0.1890	0.076*	
H20B	0.5120	0.8594	0.1617	0.076*	
H20C	0.5497	0.9098	0.2656	0.076*	
H1N2	−0.074 (4)	0.996 (3)	0.297 (2)	0.041 (7)*	

### Atomic displacement parameters (Å^2^)

**Table:**

	*U*^11^	*U*^22^	*U*^33^	*U*^12^	*U*^13^	*U*^23^
S1	0.0468 (4)	0.0560 (4)	0.0375 (4)	−0.0280 (3)	−0.0046 (3)	−0.0051 (3)
S2	0.0395 (4)	0.0391 (4)	0.0376 (4)	−0.0113 (3)	−0.0126 (3)	−0.0002 (3)
N1	0.0316 (12)	0.0483 (13)	0.0363 (12)	−0.0134 (10)	−0.0105 (10)	−0.0029 (10)
N2	0.0327 (10)	0.0388 (11)	0.0364 (11)	−0.0153 (9)	−0.0139 (9)	0.0063 (9)
C1	0.0348 (13)	0.0469 (14)	0.0313 (12)	−0.0162 (11)	−0.0185 (10)	0.0057 (11)
C2	0.0387 (14)	0.0693 (19)	0.0419 (15)	−0.0270 (14)	−0.0184 (12)	0.0059 (14)
C3	0.0574 (18)	0.074 (2)	0.0569 (18)	−0.0425 (16)	−0.0260 (15)	0.0090 (16)
C4	0.0627 (18)	0.0535 (17)	0.0560 (18)	−0.0304 (15)	−0.0293 (15)	0.0023 (14)
C5	0.0430 (14)	0.0469 (15)	0.0384 (14)	−0.0177 (12)	−0.0156 (12)	0.0000 (12)
C6	0.0358 (13)	0.0453 (14)	0.0308 (12)	−0.0165 (11)	−0.0170 (10)	0.0064 (11)
C7	0.0432 (14)	0.0409 (14)	0.0364 (13)	−0.0159 (12)	−0.0178 (11)	0.0052 (11)
C8	0.0445 (14)	0.0331 (13)	0.0408 (14)	−0.0154 (11)	−0.0174 (12)	0.0044 (11)
C9	0.0398 (13)	0.0372 (13)	0.0390 (13)	−0.0204 (11)	−0.0197 (11)	0.0088 (10)
C10	0.0369 (13)	0.0393 (13)	0.0418 (14)	−0.0210 (11)	−0.0184 (11)	0.0087 (11)
C11	0.0326 (12)	0.0320 (12)	0.0383 (13)	−0.0172 (10)	−0.0133 (10)	0.0072 (10)
C12	0.0349 (12)	0.0353 (13)	0.0413 (13)	−0.0195 (11)	−0.0174 (11)	0.0110 (10)
C13	0.0477 (15)	0.0553 (17)	0.0391 (14)	−0.0284 (13)	−0.0179 (12)	0.0103 (12)
C14	0.0640 (18)	0.0623 (18)	0.0543 (17)	−0.0386 (16)	−0.0378 (15)	0.0260 (15)
C15	0.0553 (17)	0.0438 (16)	0.078 (2)	−0.0231 (14)	−0.0432 (17)	0.0252 (15)
C16	0.0408 (14)	0.0344 (14)	0.0639 (18)	−0.0099 (11)	−0.0245 (13)	0.0071 (12)
C17	0.0394 (13)	0.0317 (12)	0.0418 (14)	−0.0178 (11)	−0.0173 (11)	0.0071 (10)
C18	0.084 (2)	0.062 (2)	0.068 (2)	−0.0288 (17)	−0.0527 (19)	0.0217 (16)
C19	0.0645 (18)	0.0451 (15)	0.0504 (16)	−0.0318 (14)	−0.0289 (14)	0.0102 (12)
C20	0.0461 (15)	0.0541 (17)	0.0544 (17)	−0.0305 (14)	−0.0179 (13)	0.0146 (13)

### Geometric parameters (Å, º)

**Table:**

S1—C6	1.756 (3)	C9—C19	1.535 (3)
S1—C7	1.856 (2)	C9—C10	1.555 (3)
S2—C17	1.728 (3)	C10—C11	1.491 (3)
S2—C11	1.746 (2)	C10—H10A	0.9700
N1—C1	1.384 (3)	C10—H10B	0.9700
N1—C7	1.464 (3)	C12—C13	1.394 (3)
N1—H1N2	0.79 (3)	C12—C17	1.394 (3)
N2—C11	1.299 (3)	C13—C14	1.374 (4)
N2—C12	1.392 (3)	C13—H13A	0.9300
C1—C2	1.384 (4)	C14—C15	1.381 (4)
C1—C6	1.400 (3)	C14—H14A	0.9300
C2—C3	1.385 (4)	C15—C16	1.372 (4)
C2—H2B	0.9300	C15—H15A	0.9300
C3—C4	1.374 (4)	C16—C17	1.391 (4)
C3—H3A	0.9300	C16—H16A	0.9300
C4—C5	1.378 (4)	C18—H18A	0.9600
C4—H4A	0.9300	C18—H18B	0.9600
C5—C6	1.380 (3)	C18—H18C	0.9600
C5—H5A	0.9300	C19—H19A	0.9600
C7—C18	1.534 (4)	C19—H19B	0.9600
C7—C8	1.534 (3)	C19—H19C	0.9600
C8—C9	1.542 (3)	C20—H20A	0.9600
C8—H8A	0.9700	C20—H20B	0.9600
C8—H8C	0.9700	C20—H20C	0.9600
C9—C20	1.532 (3)		
			
C6—S1—C7	91.74 (12)	C9—C10—H10A	108.6
C17—S2—C11	89.66 (11)	C11—C10—H10B	108.6
C1—N1—C7	114.6 (2)	C9—C10—H10B	108.6
C1—N1—H1N2	115.9 (19)	H10A—C10—H10B	107.6
C7—N1—H1N2	115.8 (19)	N2—C11—C10	125.0 (2)
C11—N2—C12	111.0 (2)	N2—C11—S2	115.04 (18)
C2—C1—N1	127.2 (2)	C10—C11—S2	119.97 (18)
C2—C1—C6	119.2 (2)	N2—C12—C13	125.6 (2)
N1—C1—C6	113.5 (2)	N2—C12—C17	115.1 (2)
C1—C2—C3	119.2 (2)	C13—C12—C17	119.4 (2)
C1—C2—H2B	120.4	C14—C13—C12	118.8 (3)
C3—C2—H2B	120.4	C14—C13—H13A	120.6
C4—C3—C2	121.3 (3)	C12—C13—H13A	120.6
C4—C3—H3A	119.4	C13—C14—C15	121.3 (3)
C2—C3—H3A	119.4	C13—C14—H14A	119.3
C3—C4—C5	120.2 (3)	C15—C14—H14A	119.3
C3—C4—H4A	119.9	C16—C15—C14	121.1 (3)
C5—C4—H4A	119.9	C16—C15—H15A	119.4
C4—C5—C6	119.2 (2)	C14—C15—H15A	119.4
C4—C5—H5A	120.4	C15—C16—C17	117.9 (3)
C6—C5—H5A	120.4	C15—C16—H16A	121.0
C5—C6—C1	120.9 (2)	C17—C16—H16A	121.0
C5—C6—S1	128.0 (2)	C16—C17—C12	121.5 (2)
C1—C6—S1	111.07 (18)	C16—C17—S2	129.3 (2)
N1—C7—C18	111.0 (2)	C12—C17—S2	109.22 (18)
N1—C7—C8	110.4 (2)	C7—C18—H18A	109.5
C18—C7—C8	108.3 (2)	C7—C18—H18B	109.5
N1—C7—S1	103.03 (17)	H18A—C18—H18B	109.5
C18—C7—S1	108.93 (19)	C7—C18—H18C	109.5
C8—C7—S1	115.00 (18)	H18A—C18—H18C	109.5
C7—C8—C9	124.5 (2)	H18B—C18—H18C	109.5
C7—C8—H8A	106.2	C9—C19—H19A	109.5
C9—C8—H8A	106.2	C9—C19—H19B	109.5
C7—C8—H8C	106.2	H19A—C19—H19B	109.5
C9—C8—H8C	106.2	C9—C19—H19C	109.5
H8A—C8—H8C	106.4	H19A—C19—H19C	109.5
C20—C9—C19	108.4 (2)	H19B—C19—H19C	109.5
C20—C9—C8	110.9 (2)	C9—C20—H20A	109.5
C19—C9—C8	106.2 (2)	C9—C20—H20B	109.5
C20—C9—C10	111.5 (2)	H20A—C20—H20B	109.5
C19—C9—C10	109.0 (2)	C9—C20—H20C	109.5
C8—C9—C10	110.70 (19)	H20A—C20—H20C	109.5
C11—C10—C9	114.51 (19)	H20B—C20—H20C	109.5
C11—C10—H10A	108.6		
			
C7—N1—C1—C2	−163.1 (2)	C7—C8—C9—C10	−46.0 (3)
C7—N1—C1—C6	20.5 (3)	C20—C9—C10—C11	61.9 (3)
N1—C1—C2—C3	−174.7 (2)	C19—C9—C10—C11	−57.7 (3)
C6—C1—C2—C3	1.5 (4)	C8—C9—C10—C11	−174.1 (2)
C1—C2—C3—C4	−0.3 (4)	C12—N2—C11—C10	−178.0 (2)
C2—C3—C4—C5	−0.8 (4)	C12—N2—C11—S2	0.9 (3)
C3—C4—C5—C6	0.7 (4)	C9—C10—C11—N2	89.5 (3)
C4—C5—C6—C1	0.5 (4)	C9—C10—C11—S2	−89.4 (2)
C4—C5—C6—S1	178.4 (2)	C17—S2—C11—N2	−1.15 (19)
C2—C1—C6—C5	−1.6 (4)	C17—S2—C11—C10	177.85 (19)
N1—C1—C6—C5	175.1 (2)	C11—N2—C12—C13	179.4 (2)
C2—C1—C6—S1	−179.83 (19)	C11—N2—C12—C17	−0.1 (3)
N1—C1—C6—S1	−3.1 (3)	N2—C12—C13—C14	−179.8 (2)
C7—S1—C6—C5	171.4 (2)	C17—C12—C13—C14	−0.4 (4)
C7—S1—C6—C1	−10.53 (19)	C12—C13—C14—C15	0.6 (4)
C1—N1—C7—C18	90.3 (3)	C13—C14—C15—C16	0.0 (4)
C1—N1—C7—C8	−149.5 (2)	C14—C15—C16—C17	−0.7 (4)
C1—N1—C7—S1	−26.2 (2)	C15—C16—C17—C12	0.9 (4)
C6—S1—C7—N1	19.83 (17)	C15—C16—C17—S2	−179.3 (2)
C6—S1—C7—C18	−98.1 (2)	N2—C12—C17—C16	179.1 (2)
C6—S1—C7—C8	140.07 (19)	C13—C12—C17—C16	−0.4 (4)
N1—C7—C8—C9	77.0 (3)	N2—C12—C17—S2	−0.8 (3)
C18—C7—C8—C9	−161.2 (2)	C13—C12—C17—S2	179.76 (19)
S1—C7—C8—C9	−39.1 (3)	C11—S2—C17—C16	−178.8 (3)
C7—C8—C9—C20	78.3 (3)	C11—S2—C17—C12	1.02 (18)
C7—C8—C9—C19	−164.2 (2)		

### Hydrogen-bond geometry (Å, º)

**Table:**

*D*—H···*A*	*D*—H	H···*A*	*D*···*A*	*D*—H···*A*
N1—H1*N*2···N2^i^	0.79 (3)	2.35 (3)	3.130 (3)	170 (4)
C10—H10*B*···S1	0.97	2.87	3.543 (2)	128
C20—H20*B*···S1	0.96	2.67	3.331 (4)	126

Symmetry code: (i) −*x*, −*y*+2, −*z*+1.
